# Exercise Intervention in Women with Fibromyalgia and Its Influence on Pain, Psychological Variables, and Disability: An Observational Study

**DOI:** 10.3390/life15010040

**Published:** 2024-12-31

**Authors:** María Elena González-Álvarez, Víctor Riquelme-Aguado, Giacomo Rossettini, Josué Fernández-Carnero, Jorge Hugo Villafañe

**Affiliations:** 1 Escuela Internacional de Doctorado, Rey Juan Carlos University, 28008 Madrid, Spain; 2 Cognitive Neuroscience, Pain, and Rehabilitation Research Group (NECODOR), Faculty of Health Sciences, Rey Juan Carlos University, 28922 Madrid, Spain; josue.fernandez@urjc.es; 3 Health Science, UNIE University, 28015 Madrid, Spain; 4 Department of Basic Health Sciences, Rey Juan Carlos University, 28933 Madrid, Spain; victor.riquelme@urjc.es; 5 Grupo de Investigación Consolidado de Bases Anatómicas, Moleculares y del Desarrollo Humano de la Universidad Rey Juan Carlos (GAMDES), 28922 Alcorcon, Spain; 6 Fisioterapia Oreka CB, 45200 Illescas, Spain; 7 Musculoskeletal Pain and Motor Control Research Group, Faculty of Sport Sciences, Universidad Europea de Madrid, 28670 Villaviciosa de Odon, Spain; mail@villafane.it; 8 Department of Physiotherapy, Faculty of Sport Sciences, Universidad Europea de Madrid, 28670 Villaviciosa de Odon, Spain; 9 Department of Physical Therapy, Occupational Therapy, Rehabilitation and Physical Medicine, Rey Juan Carlos University, 28032 Madrid, Spain

**Keywords:** fibromyalgia, telerehabilitation, physical exercise, pressure pain threshold (PPT), conditioned pain modulation (CPM), fibromyalgia impact questionnaire (FIQ)

## Abstract

(1) Background: Fibromyalgia syndrome (FM) is a specific condition within the spectrum of musculoskeletal pain disorders, with an estimated global prevalence of 2%. Physical exercise has shown promise in modulating pain and improving physical function without the drawbacks of pharmacotherapy. This study aims to examine the effects of a 6-week telerehabilitation combined exercise program—including mobility, strength, and high-intensity exercises—on pain, psychological variables, and disability in women with fibromyalgia. (2) Methods: In this observational study involving 53 FM patients, the outcomes measured were the pressure pain threshold (PPT), the conditioned pain modulation (CPM) paradigm, levels of pain on the measurement day and the average of the last week (using NRS) the impact of the fibromyalgia (using Fibromyalgia Impact Questionnaire (FIQ), and anxiety (using the Spanish version of the State–Trait Anxiety Inventory—STAI). (3) Results: Statistically significant changes were observed in the intervention group in PPT, CPM, NRS, and FIQ. (4) Conclusions: A six-week telerehabilitation therapeutic exercise intervention consisting of two scheduled exercise sessions per week lasting approximately 45 min each is associated with reduced pain levels, enhanced pain inhibitory pathways, and a decreased impact of fibromyalgia compared to patients who do not adopt a more active lifestyle.

## 1. Introduction

Chronic musculoskeletal pain represents the leading cause of disability and is a major contributor to a reduced quality of life, affecting approximately 10% of the global population [1,2]. Fibromyalgia syndrome (FM), a condition within this category, has a worldwide prevalence of approximately 2%. Although its origins are not fully understood, emerging evidence points to impairments in pain-inhibitory mechanisms as a core feature of FM’s pathophysiology [3]. Additionally, FM has recently been linked to elevated mortality rates, particularly those related to deaths due to accidents, infections, and suicide [4]. Pain modulation in FM is influenced by various factors, including alterations in neuroplasticity and dysregulation of neurotransmitter systems, such as serotonin and norepinephrine [5]. Similar mechanisms are observed in other chronic pain disorders, such as whiplash-associated disorders and mechanical neck pain, where myofascial trigger points perpetuate musculoskeletal pain through central sensitization [6]. Due to the impact of FM on quality of life, identifying effective therapeutic strategies is a must. While pharmacological treatments may provide some symptom relief [7], they are often ineffective and associated with adverse side effects, strengthening interest in nonpharmacological interventions like physical exercise.

Psychological factors play a major role in fibromyalgia, with stress being a crucial mediator between these factors and key symptoms such as pain, fatigue, and sleep disturbances [8]. Catastrophizing, anxiety, and depression are recognized as key psychological variables influencing the severity of FM and treatment outcomes [8,9]. Catastrophizing, a cognitive bias characterized by excessive negative perceptions of pain, has been identified as a predictor of mental health comorbidities, including anxiety and depression, which, in turn, lead to maladaptive coping mechanisms and poorer therapeutic responses [10].

Emerging treatment modalities, such as telerehabilitation, have shown promise in addressing both the physical and psychological dimensions of FM. This approach, which integrates exercise and pain management education delivered remotely, has demonstrated significant improvements in physical functionality, symptom severity, and reductions in pain catastrophizing in FM patients, particularly women [11]. Additionally, telerehabilitation has been associated with improvements in pain intensity, mechanical sensitivity, psychological well-being, and overall quality of life [12,13].

Physical exercise is a cornerstone in the nonpharmacological management of FM, offering numerous benefits, such as improved pain modulation and enhanced physical functionality, without the adverse effects commonly associated with pharmacotherapy [14,15,16]. Neurophysiological adaptations to exercise include enhanced neuroplasticity, increased release of pain-modulating neurotransmitters (e.g., endorphins), and reductions in systemic inflammation [17]. Exercise also contributes to improved sleep quality, reduced fatigue, and enhanced mood, collectively diminishing pain perception in FM patients. Among exercise modalities, aerobic and resistance training have demonstrated superior efficacy compared to flexibility and balance exercises [18,19,20]. However, variables such as intensity, frequency, session duration, program length, and exercise modality must be tailored to the fluctuating symptoms of FM, thus complicating exercise prescription [21,22]. Symptoms, such as chronic fatigue, which affects up to 80% of women with FM, and psychological factors like anxiety further challenge adherence to exercise programs [23,24]. This underscores the importance of individualized and adaptable exercise protocols. Combined exercise programs, which integrate the benefits of various modalities, hold promise in addressing these challenges by leveraging the synergistic effects of different neurophysiological mechanisms. However, the optimal parameters for such programs remain unclear, particularly for women with FM, due to the heterogeneity of clinical presentations and methodological variability in previous studies [22,25,26].

Moreover, existing research is inconsistent in evaluating outcomes, employing either subjective pain scales or objective assessments of somatosensory pain mechanisms [3,27,28]. Evidence remains limited regarding the effects of specific exercise modalities, such as high-intensity interval training and mobility exercises, on somatosensory pain mechanisms in FM [29,30]. To date, no study has investigated the effects of a combined program incorporating high-intensity interval training, strength exercises, and mobility exercises on clinical outcomes in FM women. The impact of combined exercise programs on clinical outcomes in women with FM remains insufficiently explored.

This study aims to examine the effects of a 6-week telerehabilitation combined exercise program—including mobility, strength, and high-intensity exercises—on pain, psychological variables, and disability in women with fibromyalgia. This program will positively influence clinical pain aspects related to central sensitization, such as mechanical hyperalgesia and conditioned pain modulation, while enhancing disability, quality of life, and anxiety symptoms. We expected that a tailored exercise program would improve their quality of life and continue to contribute to telerehabilitation as a valid, safe, and cost-effective proposal.

## 2. Materials and Methods

### 2.1. Study Design

This observational substudy is part of a controlled trial registered under ClinicalTrials.gov identifier NCT05932433 and approved by the Ethics Committee of Rey Juan Carlos University (number: 1601202303523). Participant recruitment took place during August and September 2023, and the study was conducted in the Community of Madrid between October and December 2023. All participants provided written informed consent after receiving detailed oral and written explanations about the study procedures, following the STROBE guidelines for observational studies within controlled trials [31].

### 2.2. Sample Size Calculation

In a previous study [32], patients with chronic pain demonstrated a mean improvement of 3.3 points on the visual analog scale (VAS), which ranges from 1 to 10, four weeks post-treatment. A reduction of 2 cm on the VAS is considered the minimum clinically important difference. For the study design, a power analysis was conducted with a power of 80% and a significance level of 5%. The expected mean change in the VAS score for the experimental group was set at 2, with a standard deviation of 0.4, while the control group was anticipated to have a change of less than 2. Based on these parameters, a sample size of 25 patients per group was determined to be necessary. To account for the potential loss of patients, the planned recruitment was increased to 60 patients, with 30 patients allocated to each group.

### 2.3. Participants

Due to the gender prevalence of fibromyalgia, more than 9 out of 10 patients are women [33,34], and only female patients participated in this study. They were included if they had been diagnosed with fibromyalgia, were 18 years of age or older, and were Spanish speakers. They were excluded if they had had or were having cancer, psychiatric disorders, other major illnesses (Lyme or hepatitis), irritable bowel, or diabetes.

### 2.4. Procedure and Intervention

**Figure 1:**
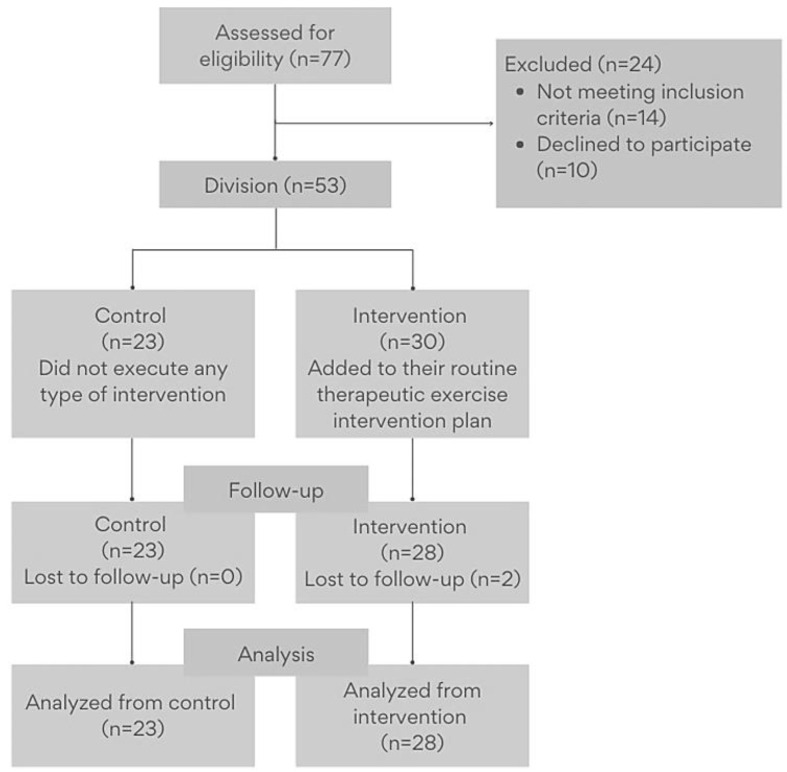
Flowchart.

Seventy-seven women with fibromyalgia showed interest in participating in this study. They were recruited through flyers, social networks, and patient associations. After preliminary screening, 14 people were excluded because they had exclusion criteria, and 10 people finally refused to participate in the study. Participants chose whether they wanted to participate in the control group (*n* = 23) or the intervention group (*n* = 30). Two patients in the intervention group could not be followed up. Figure 1 shows a flowchart of the recruiting phase. The physiotherapist was blinded to group membership and evaluated before and after the intervention.

The therapeutic exercise intervention plan included a first part of whole-body mobility warmup, a second part focused on strength (2 upper body and 2 lower body exercises, with 3 series of 10 repetitions each), and a final aerobic part involving high-intensity interval training (HIIT) lasting 4 min (20 s of intense workout and 10 s of resting until the 4 min was completed). The scientific literature agrees that active therapies, such as aerobic and strength training, are safe and effective options for fibromyalgia patients [35]. The patients were offered three different levels of exercise and were asked to choose one according to the level of pain experienced on that day. Therefore, no minimum fitness level was required, as level 1 of 3 could be performed while seated. The whole routine lasted between 35 and 45 min, considering that participants could rest as much as they needed between exercises. The material used for the entire routine was a chair and an elastic band. The whole session was recorded and sent to the patients to carry out the routine at least twice a week for 6 weeks at home. These parameters—length of routine, home-based, more than 4 weeks, and the combination of exercises—have been shown to be appropriate parameters for designing the routine [36]. Follow-up and motivational messages were sent weekly to the participants to improve treatment adherence [37]. The control group was asked to continue with their normal life without adding any extra variables related to physical exercise or healthy lifestyle habits.

Participants were assessed in the Active Recovery Madrid (Spain) physiotherapy center before and after the intervention or 6 weeks in between in the control group.

### 2.5. Outcome Measures

#### 2.5.1. Anthropometrics and Baseline Measures

Participants were asked about their age, height, weight, years since the diagnosis of fibromyalgia, pain today, and mean pain during the previous week.

#### 2.5.2. Pain Measures

**Figure 2:**
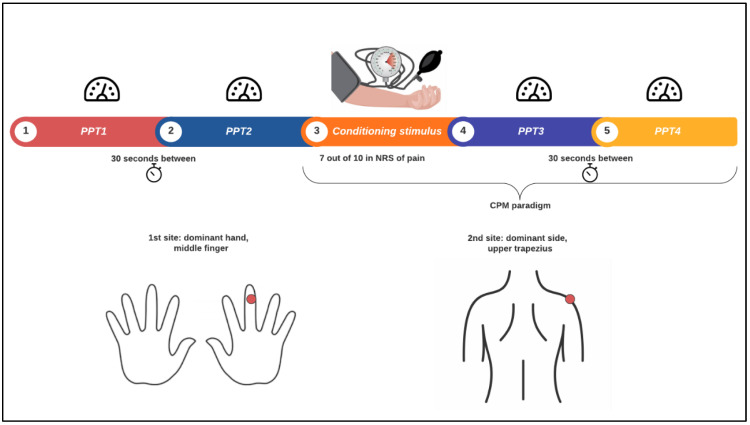
Experimental pain measurement procedure. PPT: pain pressure threshold; CPM: conditioned pain modulation; NRS: numerical rating scale.

Assessments of pain levels were conducted by a physiotherapist in a clinical setting, using an algometer (Wagner, Model FPX25, Connecticut, USA) and an inflated cuff. Participants sat on a chair with both hands on the desk. Figure 2 describes the pain-measurement protocol, which was used in a previous observational study of fibromyalgia patients [3].

The pressure pain threshold (PPT) is defined as the minimum intensity of a pressure stimulus that is perceived as painful. The PPT was measured on the middle phalanx of the dominant hand, on the middle finger. The procedure involved applying increasing pressure at 1 kg/s velocity until the subject reported the first sensation of pain. This measurement was performed twice, with a 30-s interval between each assessment. PPT measured with an algometer has been validated to assess mechanical hyperalgesia (ICC = 0.88) [38].

A pressure cuff inflated on the nondominant arm was utilized as a conditioning stimulus in the conditioned pain modulation (CPM) paradigm. The cuff was inflated with a constant pressure of 240 mm hg applied until a feeling of 7 out of 10 on a scale of pain. Afterwards, PPT was assessed again in the same way as mentioned before.

The PPT and the CPM paradigm were also assessed in the upper fibers of the trapezius, in the same circumstances described before for the middle finger [39]. The total outcome of the CPM was assessed by calculating the mean of the PPT’s postconditioning stimulus minus the mean of the PPT’s preconditioning stimulus. Positive values of the CPM total outcome indicated an elevation of the pain threshold and an efficient response of the inhibitory system.

#### 2.5.3. The Brief Pain Inventory (BPI)

The Brief Pain Inventory (BPI) is a widely used self-reported questionnaire designed to assess the impact of pain on daily life. This tool, which has been validated in research, uses a numerical rating scale (NRS) ranging from 0 to 10 to measure both pain intensity (sensory dimension) and the extent to which pain interferes with the patient’s activities and quality of life (reactive dimension). It also gathers information on pain relief, characteristics of the pain, and the patient’s perceived cause of the pain. For this study, a validated Spanish version of the BPI was employed [40,41]. Pain severity based on the NRS was classified into three categories: mild (1–5), moderate (6), and severe (7–10) [42,43]. Using the same NRS, participants were also asked about their pain during the previous week.

#### 2.5.4. Fibromyalgia Impact Questionnaire (FIQ)

The FIQ questionnaire was designed to assess the physical and psychological symptoms of FM. Scores calculate the impact of FM on daily activities and quality of life. The initial items employ a 4-point Linkert scale to evaluate physical and daily activities during the last week. The second part evaluates the feeling of well-being and work absenteeism. Seven final questions are about the disability of work, psychological distress, the severity of pain, fatigue, and muscular stiffness, from a 0 to 10 visual analogue scale, where 10 represents the maximum impact. The higher the score, the greater the impact of fibromyalgia on these patients. The Spanish version of the FIQ has good internal consistency and validity for assessing FM patients (ICC > 0.70) [41].

#### 2.5.5. Anxiety

The Spanish version of the State–Trait Anxiety Inventory (STAI) was employed to assess both trait and state anxiety. This instrument comprises 40 items in total, with 20 items dedicated to evaluating trait anxiety and 20 items for measuring state anxiety. Each item is rated on a 4-point scale. The total scores for each subscale range from 0 to 80, with higher scores indicating greater levels of anxiety [44,45].

### 2.6. Statistical Analysis

SPSS software version 29.0 (SPSS Inc., Chicago, IL, USA) was used for the statistical analysis. The demographics, anthropometric measures, pain, and clinical characteristics of the study population at baseline are summarized in Table 1, expressed as means and standard deviations (SDs). The Shapiro–Wilk normality test confirmed a normal distribution of the data. The independent-sample t-test was used to assess baseline differences between groups. For repeated measures, an analysis of covariance (ANCOVA) was performed to evaluate the effects of the intervention. Baseline scores for the outcome measures were included as covariates to adjust for initial differences, with time (pre- and post-intervention) as the within-subjects factor and group (experimental or control) as the between-subjects factor. The hypothesis of interest was group × time interaction. Post hoc comparisons were made with Bonferroni corrections. Between-group effect sizes were calculated using Cohen’s d coefficient. Effect sizes were classified as follows: large (d > 0.8), moderate (d ~ 0.5), small (d ~ 0.2), and negligible (d < 0.2). The statistical analysis was conducted at a 95% confidence level. A *p* value < 0.05 was considered statistically significant.

## 3. Results

### 3.1. Anthropometric, Demographic, and Clinical Baseline Characteristics

A total of 77 patients with fibromyalgia were screened for eligibility, of whom 53 women aged between 33 and 69 years met the inclusion criteria and provided informed consent to participate. The recruitment and retention of participants are summarized in Figure 1.

Baseline demographic, anthropometric, and clinical characteristics are presented in Table 1. The average age of the control group was 49 ± 8 years, with a BMI of 28.34 ± 6.41, while the intervention group was slightly older (52.10 ± 8.23 years) and had a lower BMI (25.61 ± 4.77). Disease duration was longer in the control group (12.22 ± 10.31 years) than in the intervention group (8.37 ± 6.03 years). Baseline scores for fibromyalgia impact (FIQ) were higher in the control group (75.89 ± 10.94) than in the intervention group (71.53 ± 14.77). Similarly, anxiety measures (STAI-S and STAI-T) were higher in the control group. PPT at the finger and trapezius were notably higher in the intervention group than in the control group at baseline.

### 3.2. Changes in Pain Sensitivity

All the results regarding the variables can be found in Table 2. Significant changes in pain sensitivity were observed between baseline and week 6. After adjusting for baseline scores, ANCOVA revealed that the intervention group experienced a significantly smaller reduction in PPT at the finger compared to the control group, with a statistically significant between-group difference of 2.19 (95% CI: 1.32 to 3.06, *p* < 0.001). Similar results were observed for PPT at the trapezius (1.47, 95% CI: 0.77 to 2.17, *p* = 0.002).

CPM at the trapezius and the finger improved significantly in the intervention group compared to the control group, resulting in a significant between-group difference of 1.89 (95% CI: 1.14 to 2.64 and CI:1.82 to 3.64, *p* < 0.001).

Pain intensity, measured on a numerical scale from 0 to 10, showed significant improvement in the control group for pain experienced in the preceding 24 h (CI: −1.40 to −0.48; *p* = 0.001). Mean pain scores for the past week also demonstrated improved values, although they did not reach statistical significance (CI: −0.81 (−1.31 to −0.31), *p* = 0.078).

### 3.3. Changes in Psychological Outcomes

As assessed by the FIQ, quality of life improved significantly in the intervention group, with an adjusted between-group difference of −11.16 (95% CI: −15.31 to −7.01, *p* < 0.001). Anxiety measures (STAI-S and STAI-T) showed minimal changes in both groups over the study period, with negligible effect sizes (Table 2).

## 4. Discussion

The objective of this study was to explore the effects of a 6-week telerehabilitation exercise program, combining mobility exercises, strength training, and high-intensity interval training, on pain, psychological variables, and disability in women with FM. The findings of this study indicate that the intervention effectively modulated central pain mechanisms, reduced the perceived burden of FM on daily functioning, and led to modest improvements in anxiety levels and overall quality of life. These results are consistent with previous research exploring the role of exercise in FM management.

One of the key findings of this study was the increase in PPT in the intervention group following the 6-week exercise protocol. This improvement aligns with the outcomes reported in previous investigations [46,47], where exercise interventions spanning eight weeks to three months also resulted in significant increases in PPT. Interestingly, these improvements in pain tolerance have been documented even after a single session of physical activity, such as a one-hour exercise intervention [48]. The observed increase in PPT suggests that the exercise program contributed to reduced pain sensitivity, further supporting its role in addressing central sensitization in FM patients.

As expected, CPM was significantly modified in the intervention group, enhancing the endogenous pain modulation process. Substantial evidence indicates that engaging in regular, long-term exercise can lower pain sensitivity and enhance endogenous pain control mechanisms in healthy individuals and those with chronic pain conditions [49]. CPM modulation has not been observed only in long-term exercise programs: Alsouhibani and Hoeger Bement have reported changes after just a single session of isometric exercise in FM patients [50]. Previous studies suggest that exercise not only alters pain-processing pathways, but also has the potential to modify brain functionality, with these changes being associated with clinical improvements in pain [51]. These paradigms are particularly significant, as they evaluate the central mechanisms involved in pain modulation in FM [52].

Regarding pain, there was a significant reduction in the NRS. This reduction in pain level is in accordance with other previous studies. Rubio-Zarapuz and collaborators explained a significant reduction in NRS levels with an exercise program very similar to ours that included a one-hour training session starting with a mobility warmup, followed by strength exercises, and concluding with HIIT [53]. Regarding the impact of fibromyalgia, statistically significant differences were observed between the patients who participated in the exercise intervention and those who did not. Previous studies have also shown a reduction in the impact of fibromyalgia through nonpharmacological interventions and active lifestyle approaches [54]. Therefore, such an approach constitutes one of the primary options in conservative treatment.

Improvements in anxiety (STAI-S and STAI-T) were minimal. They did not achieve statistical significance, suggesting that while exercise may positively influence physical and pain-related outcomes, its effects on psychological variables require further investigation. This aligns with the existing literature that emphasizes the multifactorial nature of anxiety in FM and the need for integrative therapeutic approaches to address psychological symptoms comprehensively. Contrary to our findings, McDowell and colleagues found positive changes in anxiety outcomes among patients with FM, reducing anxiety levels in long-term exercise programs lasting over 26 weeks [55]. Although not significant, physical exercise has proven to be useful in reducing mental health issues in this type of patient [56].

Furthermore, no specific type or dosage of exercise has been identified as the most effective or relevant for patients with chronic pain; both aerobic exercise and strength training have beneficial effects on FM symptoms [57]. This riddle of exercise type and dosage is common across other chronic pain conditions, where it has ultimately been suggested that the choice of exercises should probably depend on the preferences of the patient or therapist, as well as on cost-effectiveness considerations [58]. HIIT modality has shown previous benefits in chronic musculoskeletal disorders, reducing pain intensity and increasing VO2 max [59]. Allowing patients to choose whether they wanted to participate in an exercise intervention or not and sending them reminders and motivational messages throughout the intervention contributed to a positive strategy to encourage patients to follow the treatment. This fact is important and highlights the motivation of some patients to change their lifestyle, as other studies have emphasized the difficulty of this, achieving less than 40% adherence to exercise programs [60]. Moreover, telemedicine programs for fibromyalgia patients are safe and useful for reducing clinically meaningful effects of pain [12,61] The main risks of telerehabilitation, such as musculoskeletal pain, dizziness, and falls, are generally nonserious and infrequent. Previous studies have found no significant differences in adverse event occurrence across different modes of telerehabilitation, suggesting that it is a safe approach for rehabilitation [62].

### 4.1. Limitations of the Study

This study presents some limitations that should be considered before interpreting the results. First, participants self-selected their group (intervention or control), which may have introduced selection bias, dividing the sample into “actively predisposed” and “sedentarily predisposed” categories. This could influence baseline characteristics and potentially affect the generalizability of the findings.

Second, the sample size, although sufficient for statistical analysis, was relatively small and exclusively female, which limits the generalizability of the results to the broader population of FM patients, including males. Expanding the sample in future studies could provide a more comprehensive understanding of the effects of exercise interventions across diverse demographic groups.

Third, the intervention duration was limited to six weeks, and only one post-intervention follow-up was conducted. This restricts the ability to evaluate the sustainability of the observed improvements and their long-term impact on FM symptoms. Longer-term studies with multiple follow-ups are necessary to assess the persistence of benefits.

Fourth, while the exercise program was tailored to individual symptom levels and designed to enhance adherence, other factors, such as participants’ medications, dietary habits, and baseline physical activity levels, were not controlled. These variables could have influenced the outcomes, adding potential confounders to the interpretation of the results.

Lastly, the study did not include a healthy control group, which limits comparisons to a baseline reference population. Including such a group in future research could help clarify the specific impact of the intervention on FM-related dysfunctions versus general health improvements from exercise.

### 4.2. Future Directions

Future research should consider extending intervention durations to 8 or 12 weeks to evaluate the cumulative effects of exercise. Long-term follow-ups are necessary to assess the sustainability of outcomes and the impact of maintaining an active lifestyle. Additionally, integrating biomarker analysis could provide insights into the biological mechanisms underlying exercise-induced improvements. Lastly, research should aim to determine the optimal exercise type and dosage for FM management, tailored to individual patient profiles [63].

### 4.3. Clinical Implications

This study supports the implementation of individualized, home-based exercise programs as a safe and cost-effective approach to managing FM symptoms. The adaptability of telerehabilitation allows patients to tailor the intensity of exercises to their condition, promoting adherence and accessibility. These findings strengthen the potential of such interventions to complement pharmacological treatments and improve patient outcomes, particularly in resource-limited settings. Telerehabilitation as an alternative method for the treatment and monitoring of patients will imply paradigm shifts in the future. It will allow more patients to be reached, offer a better cost-effectiveness ratio compared to conventional therapies, and provide opportunities for patients unable to receive their treatments in healthcare centers to start high-quality rehabilitation supervised by a professional [64].

## 5. Conclusions

A six-week telerehabilitation exercise intervention consisting of two sessions per week and integrating mobility, strength, and high-intensity exercises was associated with improvements in pain modulation, reduced FM impact, and enhanced quality of life in women with FM. Telerehabilitation provides a valuable approach for women with fibromyalgia, allowing them to manage symptoms through tailored exercises and therapies in the comfort of their homes, improving adherence to treatment plans, and being an effective nonpharmacological strategy for managing FM.

## Figures and Tables

**Table 1:** Demographic, anthropometric, and clinical characteristics at baseline.

Variables	Control (Mean ± SD)	Intervention (Mean ± SD)	Shapiro–Wilk Normality Test (*p*-Value)
Age (years)	49.44 ± 8.63	52.10 ± 8.23	C: *p* = 0.209; I: *p* = 0.226
Height (cm)	162.87 ± 5.86	162.6 ± 6.95	C: *p* = 0.063; I: *p* = 0.854
Weight (kg)	74.78 ± 15.04	67.61 ± 12.55	C: *p* = 0.994; I: *p* = 0.407
BMI (kg/m^2^)	28.34 ± 6.41	25.61 ± 4.77	C: *p* = 0.425; I: *p* = 0.930
Years with FM	12.22 ± 10.31	8.37 ± 6.03	C: *p* = 0.711; I: *p* = 0.761
NRS 24 h	7.13 ± 1.39	6.90 ± 1.62	C: *p* = 0.285; I: *p* = 0.244
NRS 1 w	7.78 ± 1.70	7.20 ± 1.79	C: *p* = 0.321; I: *p* = 0.591
FIQ	75.89 ± 10.94	71.53 ± 14.77	C: *p* = 0.750; I: *p* = 0.726
STAI-S	38.61 ± 8.21	34.20 ± 13.34	C: *p* = 0.141; I: *p* = 0.165
STAI-T	36.30 ± 7.69	33.73 ± 12.29	C: *p* = 0.814; I: *p* = 0.512
PPT—finger	3.78 ± 2.08	5.55 ± 3.10	C: *p* = 0.988; I: *p* = 0.793
PPT—trapezius	2.46 ± 1.18	3.47 ± 2.50	C: *p* = 0.085; I: *p* = 0.548

BMI: Body Mass Index; NRS: Numerical Rate Scale; FIQ: Fibromyalgia Impact Questionnaire; STAI-S: State-Trait Anxiety Inventory—State; STAI-T: State–Trait Anxiety Inventory—Trait; PPT: Pressure Pain Threshold; C: Control; I: Intervention.

**Table 2:** Adjusted means and differences in clinical outcomes post-intervention using ANCOVA.

Outcome	Group	Adjusted Post-Mean ± SE	Adjusted Difference Within Groups	Adjusted Difference Between Groups
PPT Finger	Intervention	4.59 ± 0.25	−0.96 (−1.15 to −0.77)	2.19 * (−1.32 to 3.06)
	Control	2.41 ± 0.20	−1.37 (−1.56 to −1.18)	
PPT Trapezius	Intervention	2.92 ± 0.35	−0.55 (−1.01 to −0.09)	1.47 (−0.77 to 2.17)
	Control	1.45 ± 0.25	−1.01 (−1.22 to −0.80)	
CPM Trapezius	Intervention	3.14 ± 0.30	0.72 (−1.27 to 1.71)	1.89 * (−1.14 to 2.64)
	Control	1.25 ± 0.20	−0.93 (−1.38 to −0.48)	
CPM Finger	Intervention	5.03 ± 0.20	−0.12 (−1.33 to 1.09)	2.73 * (1.82 to 3.64)
	Control	2.30 ± 0.97	−0.92 (−1.67 to −0.17)	
FIQ	Intervention	64.98 ± 1.42	−6.55 (−12.07 to −1.03)	−11.16 * (−15.31 to −7.01)
	Control	76.14 ± 1.08	0.35 (−4.29 to 4.99)	
NRS 24 h	Intervention	6.53 ± 1.80	−0.37 (−0.98 to 0.24)	−0.94 * (−1.40 to −0.48)
	Control	7.48 ± 1.40	0.38 (−0.25 to 1.01)	
NRS 1 w	Intervention	6.67 ± 1.58	−0.53 (−1.20 to 0.14)	−0.81 (−1.31 to −0.31)
	Control	7.47 ± 1.72	−0.14 (−0.87 to 0.59)	
STAI-S	Intervention	34.40 ± 9.11	0.20 (−4.78 to 5.18)	−5.12 (−8.87 to −1.37)
	Control	39.52 ± 11.48	−0.05 (−3.48 to 3.38)	
STAI-T	Intervention	41.80 ± 11.16	8.07 (3.48 to 12.66)	−4.25 (−7.71 to −0.79)
	Control	46.05 ± 11.28	9.00 (5.75 to 12.25)	

PPT: Pressure Pain Threshold; CPM: Conditioned Pain Modulation; NRS: Numerical Rate Scale; FIQ: Fibromyalgia Impact Questionnaire; STAI-S: State–Trait Anxiety Inventory—State; STAI-T: State–Trait Anxiety Inventory—Trait. * Statistically significant (*p* < 0.05). Adjusted post-means and differences were calculated using ANCOVA, with baseline scores as covariates. Values are presented as adjusted means ± standard error (SE) or as mean differences with 95% confidence intervals.

## Data Availability

The original contributions presented in this study are included in the article. Further inquiries can be directed to the corresponding authors.
